# Phosphosulindac (OXT-328) prevents and reverses chemotherapy induced peripheral neuropathy in mice

**DOI:** 10.3389/fnins.2023.1240372

**Published:** 2024-01-29

**Authors:** Aryah Basu, Jennifer Y. Yang, Vasiliki E. Tsirukis, Andrew Loiacono, Gina Koch, Ishan A. Khwaja, Mahila Krishnamurthy, Nicholas Fazio, Emily White, Aayushi Jha, Shrila Shah, Cameron Takmil, Deniz Bagdas, Aylin Demirer, Adam Master, Ernest Natke, Robert Honkanen, Liqun Huang, Basil Rigas

**Affiliations:** ^1^Departments of Preventive Medicine, Stony Brook University, Stony Brook, NY, United States; ^2^Department of Psychiatry, Yale School of Medicine, New Haven, CT, United States; ^3^Department of Cancer Biology, Mayo Clinic, Jacksonville, FL, United States; ^4^Departments of Ophthalmology, Stony Brook University, Stony Brook, NY, United States; ^5^Medicon Pharmaceuticals, Inc, Setauket, NY, United States

**Keywords:** chemotherapy-induced peripheral neuropathies (CIPN), phosphosulindac, neuroinflammation, paclitaxel, mechanical allodynia, prevention/treatment of CIPN

## Abstract

**Background:**

Chemotherapy-induced peripheral neuropathy (CIPN), a side effect of chemotherapy, is particularly difficult to treat. We explored whether phosphosulindac (PS), a modified NSAID, could treat CIPN.

**Methods:**

CIPN was induced in male C57BL/6 J mice by paclitaxel, vincristine or oxaliplatin. Mechanical allodynia was measured with the von Frey test and cold allodynia with the acetone test. To determine the preventive effect of PS, it was administered 2 days before the induction of CIPN. Mouse Lewis lung carcinoma xenografts were used to determine if PS altered the chemotherapeutic efficacy of paclitaxel. Cultured cell lines were used to evaluate the effect of PS on neuroinflammation.

**Results:**

Treatment with each of the three chemotherapeutic agents used to induce CIPN lowered the mechanical allodynia scores by 56 to 85% depending on the specific agent. PS gel was applied topically 3x/day for 16–22 days to the hind paws of mice with CIPN. This effect was dose-dependent. Unlike vehicle, PS returned mechanical allodynia scores back to pre-CIPN levels. PS had a similar effect on paclitaxel-induced CIPN cold allodynia. Sulindac, a metabolite of PS, had no effect on CIPN. PS significantly prevented CIPN compared to vehicle. Given concomitantly with paclitaxel to mice with lung cancer xenografts, PS relieved CIPN without affecting the anticancer effect of paclitaxel. The enantiomers of PS were equally efficacious against CIPN, suggesting the therapeutic suitability of the racemate PS. There were no apparent side effects of PS. PS suppressed the levels of IL-6, IL-10, CXCL1, and CXCL2 induced by paclitaxel in a neuroblastoma cell line, and macrophage activation to the M1 proinflammatory phenotype.

**Conclusion:**

Topically applied PS demonstrated broad therapeutic and preventive efficacy against CIPN, preserved the anticancer effect of paclitaxel, and was safe. Its anti-CIPN effect appears to be mediated, in part, by suppression of neuroinflammation. These data support further evaluation of topical PS for the control of CIPN.

## 1. Introduction

Chemotherapy-induced peripheral neuropathy (CIPN), a side effect of chemotherapy, has a prevalence as high as 85%, depending on its duration and the agent used: 72.3% with oxaliplatin, 42.2% with cisplatin, 70.8% with paclitaxel, 19.6% with vincristine (Seretny et al., 2014; Flatters et al., 2017).

CIPN pain is caused by damage to nerve structures such as peripheral nerve endings, which become extremely sensitive to stimulation and can generate pulses even in the absence of stimulation (Staff et al., 2017). Patients diagnosed with CIPN may report sharp pains, dull aches, paresthesia, altered sensitivity to cold, loss of proprioception, difficulty in using the hands or in walking (Kolb et al., 2016), numbness, and loss of pain sensation. Hands and feet are most typically affected.

The neuropathic pain of CIPN is particularly difficult to treat (Hou et al., 2018; Loprinzi et al., 2020). With the exception of tetrodotoxin, several agents have failed to show efficacy when subjected to the rigors of a clinical trial (Quintao et al., 2019; Huerta et al., 2023). It is currently managed with oral antidepressants (e.g., duloxetine) and/or antiepileptics (e.g., gabapentin and pregabalin), which provide unsatisfactory pain control and induce major side effects and poor treatment adherence. The only approved drug (duloxetine) is generally considered ineffective. A recent ASCO guideline states that “no agents are recommended for the prevention of CIPN” and that “duloxetine is the only agent that has appropriate evidence to support its use for patients with established painful CIPN. Nonetheless, the amount of benefit from duloxetine is limited” (Loprinzi et al., 2020).

CIPN adversely affects cancer patients: First, CIPN can have a profound negative impact on long-term quality of life (Park et al., 2013; Ewertz et al., 2015). Second, CIPN increases the cost of care (Pike et al., 2012). Third, symptoms can be so severe that patients deem CIPN so unacceptable that they choose to discontinue treatment despite the potential for decreased survival (Miltenburg and Boogerd, 2014).

The mechanism of CIPN appears complex and may vary depending on the causative agent (Eldridge et al., 2020; Kocot-Kępska et al., 2021). Possible mechanisms include the following: effects on mitochondria, which lead to oxidative stress (Doyle and Salvemini, 2021); effects on ion channels, which may alter nerve signal conduction (Aromolaran and Goldstein, 2017); disruption of microtubules, which could impact axonal transport of proteins, RNA and mitochondria along the nerve axon (Chua et al., 2022); and neuroinflammation which could enhance neuronal excitability and pain hypersensitivity (Makker et al., 2017). Neuroinflammation is emerging as a key component in the pathophysiology of CIPN, with innate and adaptive immune responses contributing to its symptoms, while macrophages and glial cells help maintain the neuroinflammatory process (Fumagalli et al., 2021). Macrophages, forming dense infiltrates in CIPN affected nerves (Ma et al., 2022), may significantly contribute to peripheral neuropathies, including CIPN (Msheik et al., 2022). Interestingly, interferon-γ can enhance macrophage activation, inducing high levels of proinflammatory cytokines and low levels of anti-inflammatory cytokines (Schroder et al., 2004).

The resultant secretion of pro-inflammatory mediators may lead to some of the symptoms of CIPN. In particular, pro-inflammatory cytokines released upon treatment are considered a key trigger of neuroinflammation in the sensory nervous system leading to sensitization of nociceptors and mechanical hypersensitivity (Brandolini et al., 2019). Among them, IL-6 plays a large role in the inflammatory process following nerve injury and has been implicated in the initiation and maintenance of neuropathic pain (De Jongh et al., 2003). It was shown, for example, that women with breast cancer and CIPN had significantly higher levels of IL-6 compared to controls (Starkweather, 2010). Anti-inflammatory cytokines such as interleukin IL-10, can reduce the neuropathic pain of CIPN. CXCL-1 and CXCL-2, both pro-inflammatory cytokines known to mediate the infiltration of neutrophils and monocytes/macrophages (Lee et al., 2018), seem to also play a significant role in CIPN (Biber and Boddeke, 2014). For example, their expression is altered in CIPN, and receptor antagonists attenuate neuropathic pain (Zhou et al., 2020).

The relationship between changes in cytokine expression and CIPN symptoms has been explored in animal models and indirectly in patients with CIPN. For example, intraperitoneally injected IL-6 induced nociceptive hypersensitivity (Cunha et al., 2005) while IL-6 knockout mice demonstrated lowered mechanical allodynia following vincristine treatment (Kiguchi et al., 2008). Animal data align with clinical studies demonstrating increased IL-6 serum levels in CIPN-affected women following chemotherapy, indicating the importance of assessing IL-6 as a potential mediator of CIPN (Starkweather, 2010). Another example is provided by CCL2, which is upregulated in injured DRG neurons implying a role as neuron-migroglia signaling factor participating in the pathogenesis of neuropathic pain (Zhang et al., 2007). Finally, IL-10 gene therapy in rats (injected with an IL-10-encoding plasmid) prevents and reverses paclitaxel-induced mechanical allodynia (Schroder et al., 2004; Ledeboer et al., 2007).

Given the lack of efficacious treatments for CIPN, we explored whether phosphosulindac (PS, OXT-328) could be efficacious in its prevention and treatment. PS consists of conventional sulindac to which a diethylphosphate group is covalently attached through a butane spacer. PS, extensively studied for its anticancer and anti-inflammatory properties (Sun and Rigas, 2008; Mackenzie et al., 2011; Huang et al., 2011a,b; Cheng et al., 2012, 2013; Mattheolabakis et al., 2013), consistently displayed higher efficacy than sulindac and lacked side effects. Its anticancer efficacy is significant and broad, encompassing colon, lung and skin cancer (applied topically in the latter (Cheng et al., 2012). PS was also efficacious in animal models of arthritis (Huang et al., 2011a; Mattheolabakis et al., 2013), essentially reversing joint inflammation and edema.

An important feature of PS is its chirality, deriving from the chiral center at its methyl sulfoxide moiety (Brunell et al., 2011). Chirality can shape the pharmacology of a given drug (Silvestri and Colbon, 2021), and differences in efficacy and toxicity can exist between enantiomers. Several NSAIDs are chiral molecules, and some are marketed as a single enantiomer. Thus, we separated the enantiomers of PS and determined their efficacy in an animal model of paclitaxel-induced peripheral neuropathy (PN).

## 2. Materials and methods

### 2.1. Cell lines

Three murine cell lines, Neuro-2a (neuroblastoma), RAW264.7 (macrophage), and Lewis lung carcinoma (LL/2), were obtained from the American Type Culture Collection (ATCC, Manassas, VA) and grown as monolayers in the culture medium and under conditions suggested by ATCC. All cell lines were passaged in our laboratory for less than 6 months after receipt.

### 2.2. Phosphosulindac, its enantiomers and their formulation

Phosphosulindac was a gift of Medicon Pharmaceuticals, Inc., Setauket, NY. We separated the two enantiomers of PS by supercritical fluid chromatography using an AD-H (25×0.46 cm) column (3,5-dimethylphenylcarbamate bound to amylose supported on silica gel; Analytics-Shop USA LP, Stockbridge GA) and purified several grams of each.

PS and its two enantiomers were each formulated in a gel based on hydroxypropyl-methylcellulose as the gelling agent and xanthan gum as a phase stabilizer. Briefly, PS was solubilized in a 4:1 ethanol: triethanolamine mixture, and this was followed by the addition of water containing the perservative benzalkonium chloride 0.05% w/v. After proper mixing, 15 mg of both xanthan gum and hydroxypropyl methylcellulose were added and the mixture was sonicated. This was followed by addition of 100 μL of glycerol and thorough mixing. Finally, the pH of the gel was adjusted to 7.5 with sodium citrate.

### 2.3. Chemotherapeutic drugs

Paclitaxel, oxaliplatin, and vincristine were purchased from MilliporeSigma (St. Louis, MO). Paclitaxel was dissolved in a mixture of 1 volume ethanol/1 volume Cremophor EL (EMD Millipore Corp, Burlington, MA)/18 volumes distilled water. Oxaliplatin was dissolved in ddH_2_O. Vincristine was dissolved in PBS. All injections were administered intraperitoneally (i.p.) in a volume of 1 mL/100 g body weight.

### 2.4. Animals

The strain, sex and age of the mice to be used in CIPN studies are critical determinants of the degree of allodynia that can be induced by specific chemotherapeutic agents [reviewed in Höke and Ray (2014), Gadgil et al. (2019)]. Younger male mice appear to display more robust allodynia in response to chemotherapy. Preliminary work with several strains of mice and other parameters led us to conduct our studies using adult male C57BL/6 J mice, 8 weeks of age at the beginning of the experiments and weighing 20–30 g (The Jackson Laboratory, Bar Harbor, ME). Mice were housed in an AAALAC-accredited facility in groups of four. Food and water were available *ad libitum*. The mice in each cage were randomly allocated to treatment groups. All studies were conducted by experimenters blinded to the identity of the treatment groups. Experiments were performed during the light cycle (7:00 am to 7:00 pm) and animals were euthanized with CO_2_ asphyxiation. Studies were approved by the Institutional Animal Care and Use Committee of Stony Brook University and followed the National Institutes of Health *Guide for the Care and Use of Laboratory Animals*. Animal studies are reported in compliance with the ARRIVE guidelines (Kilkenny et al., 2010).

### 2.5. Mouse xenograft

Adult male C57BL/6 J mice as above were inoculated subcutaneously into each flank with 1.0 × 10^6^ murine Lewis lung carcinoma cells in PBS (final volume 100 μL). Once the tumor reached approximately 180 mm^3^, animals were randomized into the control group, which received the solvent of paclitaxel, and the three treatment groups, which received only paclitaxel or paclitaxel plus vehicle gel or 5% PS gel (*n* = 8 per group). Paclitaxel 12 mg/kg was administered intraperitoneally once daily for 5 consecutive days. The vehicle or PS gel was applied topically onto both hind paws of the mice 3x/day, 7 days/week for 14 days. Tumor volume was calculated as [length × width × (length + width/2) × 0.56].

### 2.6. Induction of CIPN in mice and treatment with PS

CIPN was induced in mice with chemotherapeutic agents using established protocols (Carozzi et al., 2010; Currie et al., 2019; Eldridge et al., 2020). Dosing regiments for each of the three chemotherapeutics were as follows. *Paclitaxel:* Four i.p. injections of 8 mg/kg paclitaxel every other day, resulting in a cumulative dose of 32 mg/kg. *Oxaliplatin:* Oxaliplatin 3 mg/kg was injected i.p. daily for 5 days, followed by 5 days of no treatment, which was followed by another 5-day period of daily oxaliplatin i.p. injections as previously for 10 injections leading to a cumulative dose of 30 mg/kg. *Vincristine:* Two i.p. injections of vincristine 1.5 mg/kg were made within one week for a total cumulative dose of 3 mg/kg.

We used one protocol for the treatment of established CIPN and a second for its prevention. *Treatment protocol:* Once CIPN was established, documented by reduced mechanical allodynia threshold, PS 8% or vehicle control (gel) was applied three times daily to the hind paws of the mice for the indicated period. *Prevention protocol:* Administration of PS 8% or vehicle to the hind paws of mice as above started two days before initiating the administration of paclitaxel as above. Mechanical allodynia was measured before the administration of PS and on day 10 after initiation of PS treatment.

### 2.7. Determination of allodynia

In these mice, we determined both mechanical and cold allodynia, as described below.

#### 2.7.1. Mechanical allodynia evaluation (von Frey test)

Mechanical allodynia thresholds were determined using von Frey filaments according to an established method (Chaplan et al., 1994; Bagdas et al., 2015). Briefly, mice were placed in a quiet room for 30 min and then put in a Plexiglas cage with mesh metal flooring where they were allowed to acclimate for 15 min before testing. A series of calibrated von Frey filaments (Stoelting, Wood Dale, IL) with incremental stiffness were applied perpendicularly to the paw with sufficient force to cause slight bending and held 2–3 s. This process was repeated at each level of stiffness 5 times, a few seconds apart. Paw withdrawal, licking or shaking were considered positive responses. The mechanical threshold or the paw withdrawal threshold (PWT) expressed in g, indicates the force of the von Frey filament to which the animal reacted.

#### 2.7.2. Cold allodynia evaluation (acetone test)

This was performed according to published methods (Otrubova et al., 2013; Toma et al., 2017). After placing the mice in a quiet room and acclimating them as above, we applied 10 μL of acetone held in the end of a pipette to the plantar surface of each hind paw by a blast of air applied to the other end of the pipette. Total time spent licking, lifting and/or shaking of the hind paw during a 1-min test period was recorded as the cold allodynia score, which was measured in seconds.

### 2.8. Co-culture and determination of cytokine levels

Neuro-2A and RAW 264.7 cells were co-cultured in the presence and absence of paclitaxel and/or PS. Cells were grown in basic growth media including 1:1 mixture of DMEM (Gibco, 11,995,065) and EMEM (ATCC, 30–2003) with 10% heat-inactivated fetal bovine serum (Sigma Aldrich, F9665), 1% penicillin/streptomycin and 2 mM glutamine. Cells were split 1:10–1:20 using DPBS (Sigma, D8537) and 0.05% trypsin–EDTA (Fisher Scientific, 10,779,413) at 70–80% confluence. Only cells below passage 15 were used. For co-culture, 0.3×10^6^ cells of each cell line were seeded in 6-well plates (ThermoFisher Scientific, 165,218) and incubated overnight. Immediately before treatment, the culture medium was supplemented with DMSO (Sigma-Aldrich, D2650) for controls, or paclitaxel (Sigma-Aldrich,T7402), PS or their combination. Stock solution in DMSO (Sigma-Aldrich, D2650) of paclitaxel 10 mM and PS 100 mM were used to provide the final concentration of 1 μM and 20 μM, respectively (Stage et al., 2020). Control samples were treated with DMSO. After 24 h, media were collected, centrifuged and frozen at −80\u00B0C until analyzed.

The levels of cytokines in the cell culture medium were determined using the magnetic bead-based immunoassay kit (Luminex 200; Luminex Corp., Austin, TX) and the MILLPLEX Mouse Cytokine/chemokine Magnetic Panel (#MPXMCYTO-70 K, Millipore, Billerica, MA). Cell supernatant samples were incubated with antibody-coated capture beads overnight at 4°C. Washed beads were further incubated with biotin-labeled anti-human cytokine antibodies, followed by streptavidin– phycoerythrin incubation. Standard curves of known concentrations of recombinant mouse cytokines/chemokines were used to convert fluorescence units to concentrations (μg/mL). To calculate the molecular concentration of cytokines in culture media samples, we analyzed the median fluorescent intensity data using a 5-parameter logistic or spline curve-fitting method.

### 2.9. Macrophage activation

Murine macrophage RAW 264.7 cells were grown in DMEM (ThermoFisher Scientific, Waltham, MA) supplemented with 10% FBS, 1% penicillin–streptomycin, 0.5 mM 2-mercaptoethanol, and 40 mM HEPES buffer (all from Sigma, St. Louis, MO) at 37°C in a 5% CO_2_ incubator. Cells, maintained for <6 weeks to avoid unresponsiveness to IFN-γ and LPS, were pre-incubated for 24 h with recombinant IFN-γ and LPS from *E. coli O55:B5* (Sigma, St. Louis, MO) in Opti-MEM (Invitrogen Life Technologies, Waltham, MA) after which IFN-γ and LPS containing media were replaced with DMEM as previously. Activated RAW cells displayed morphological changes (Zhang et al., 2007). Prior to treating these cells with 20 μM PS or 1 μM paclitaxel or 20 μM PS plus 1 μM paclitaxel for 24 h, their culture medium was changed to EMEM:DMEM, 1:1 supplemented with 10% FBS.

### 2.10. Curve fitting and EC_50_ calculation

A software license for Prism 10.0.2 was purchased from Graphpad Software, LLC (Boston, MA, USA) to analyze dose–response data and to calculate the EC_50_ for phosphosulindac efficacy in restoring mechanical allodynia scores back to normal control levels prior to the administration of paclitaxel. The data were analyzed using the software’s Nonlinear fit – [Agonist] vs. response – Variable Slop (four parameter) routine. The data are fit to the equation where:


Y=Bottom+(X^Hillslope)∗(Top−Bottom)/(X^Hillslope+EC50^Hillslope).

The EC_50_ is the dose of agonist that gives a response half way between Bottom and Top. The Hill slope describes the steepness of the family of curves. A Hill slope of 1.0 is standard; a Hillslope >1.0 is steeper and a Hill slope of <1.0 is shallower.

Top and Bottom are plateaus in the units of the *Y* axis.

### 2.11. Statistical analysis

Results are expressed as mean ± SEM. All values obtained have been included in the analysis. Analysis of variance (ANOVA) tests were conducted and followed by the Bonferroni *post hoc* test. Differences were determined to be significant at *p* < 0.05/*.

## 3. Results

### 3.1. Therapeutic efficacy of PS

We assessed the effect of PS in mice with established CIPN, reflecting the clinical situation in which patients present with CIPN after their chemotherapy is completed.

**Figure 1:**
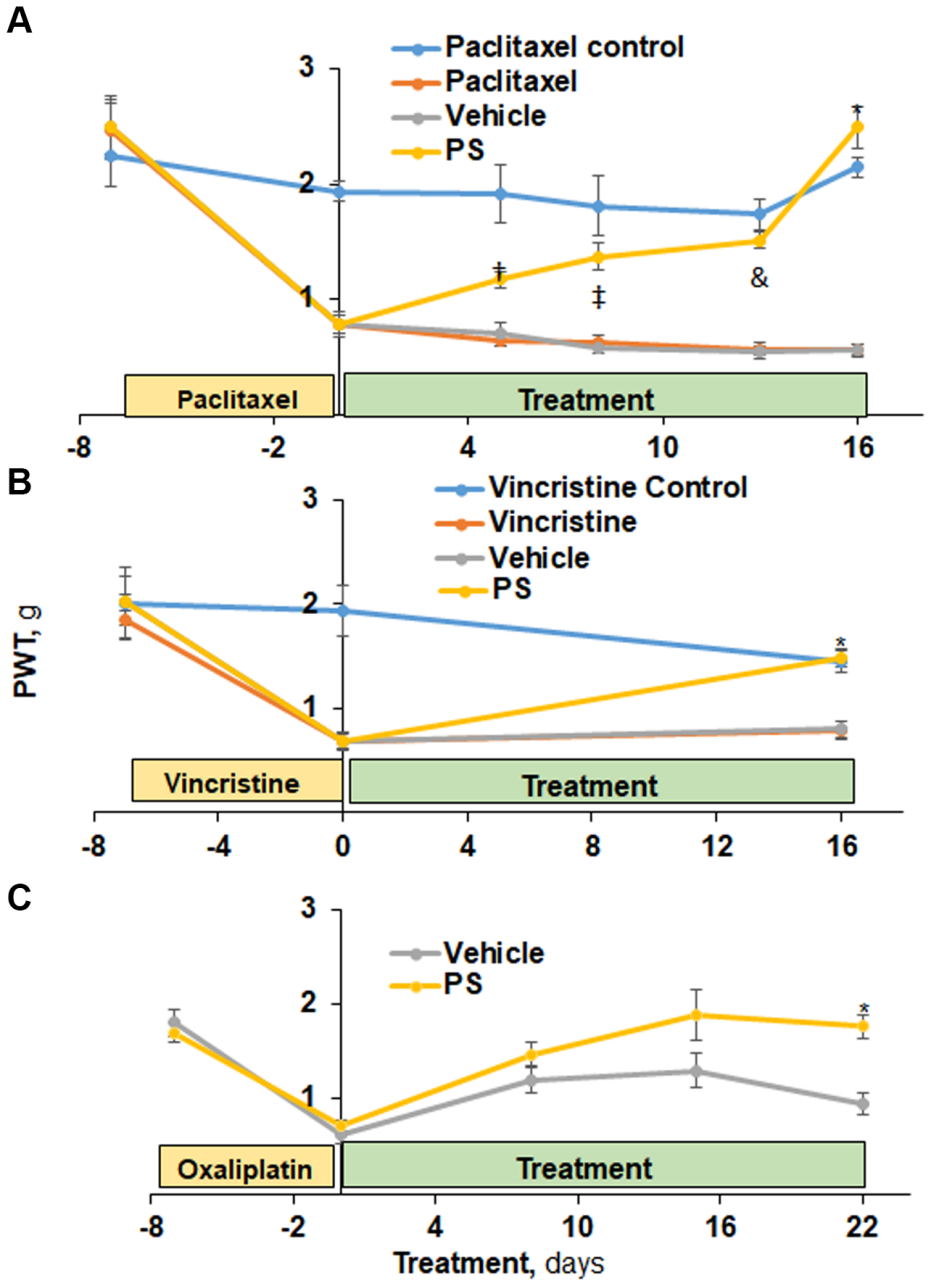
The efficacy of PS in the treatment of CIPN mechanical allodynia. **(A)** Three groups of mice were treated with paclitaxel and one with its solvent control (*n* = 6-8/group) for 7 days as in Methods for the induction of CIPN. Treatment of two of the first three groups with PS or vehicle gel applied to the hind paws 3×/d × 16 d started on day 0. Mechanical allodynia was determined manually with the von Frey test at the indicated time points and was expressed as the paw withdrawal threshold (PWT) measured in grams. ^†^*p* < 0.05, ^‡^*p* < 0.01, ^&^*p* < 0.001, *p < 0.0001. Mixed Model results: Group (*F*(3, 3) = 50.8, *p* < 0.005); Time (*F*(5, 105) = 47.7, *p* < 0.0001); Group*Time (*F*(15, 15) = 7.8, *p* = 0.0001). **(B)** A similar study was performed using vincristine to induce CIPN. Mixed Model results: Group (*F*(3, 52) = 9.04, *p* < 0.0001); Time (*F*(2, 104) = 40.2, *p* < 0.0001); Group*Time (*F*(6, 104) = 7.82, *p* = 0.0001). At day 16, the difference in mechanical allodynia scores between PS and its vehicle control is highly significant. *p < 0.0001, *n* = 7. **(C)** Two groups of mice (*n* = 8/group) were treated with PS or vehicle as above for 22 days after the induction of CIPN by oxaliplatin (days-15 to 0). Mixed Model results: Group (*F*(1, 8) = 3.94, *p* = 0.0825); Time (*F*(4, 32) = 44.19, *p* < 0.0001); Group*Time (*F*(4, 32) = 3.13, *p* = 0.05). The mechanical allodynia score at D22 for PS treated mice is more toward normal untreated baseline and significantly different from vehicle treated mice. *p < 0.001, *n* = 5. All values: *mean ± SEM*.

As shown in Figures 1A–C, each of the three anticancer drugs that we studied induced significant CIPN, evidenced by allodynia. Topical treatment with PS 8% gel 3x/day was started after the neuropathy was established. This dose was chosen from previous studies in our laboratory investigating the efficacy of PS as an anti-cancer and anti-arthritis agent (Mackenzie et al., 2010; Huang et al., 2011a).

Initially, we examined the effect of PS on paclitaxel-induced peripheral neuropathy using mechanical allodynia as the measured outcome (Figure 1A). At baseline, all four study groups of mice had similar allodynia scores (range 2.24 ± 0.26 g to 2.49 ± 0.24 g; *mean ± SEM* for this and all subsequent values). Paclitaxel administered to three study groups over 12 days reduced by ~85% their mechanical allodynia scores (*t*(15) range = 7.2–7.9, all *p* < 0.0001) from baseline to day zero indicative of CIPN while no such reduction was observed in the non-treated paclitaxel control (*t*(15) = 1.5, *p* = 0.16). In contrast, the control group (non-paclitaxel, non-PS) showed a minor, statistically non-significant variation in allodynia scores throughout the entire study period. When PS was applied to the paws of mice with paclitaxel induced PN, their allodynia score showed progressive improvement from its post-chemotherapeutic baseline at the initiation of treatment, returning it to its pre-treatment baseline on day 16 (day 0 = 0.79 ± 0.08 g vs. day 16 = 2.49 ± 0.18 g; *p* < 0.0001). The paclitaxel-only treated group showed changes in the allodynia score similar to those of the vehicle group (day 0 = 0.78 ± 0.08 g vs. day 16 = 0.57 ± 0.05 g; *p* = NS). The difference between the PS-treated group and its vehicle control first became statistically significant on day 5 (PS = 1.17 ± 0.07 g, vehicle = 0.7 ± 0.07 g; *p* = 0.05), with their difference increasing thereafter and becoming maximal on day 16 (PS = 2.49 ± 0.18 g, vehicle = 0.56 ± 0.05 g; *p* < 0.0001).

PS improved the mechanical allodynia induced by the commonly used vincristine (Skubnik et al., 2021). In the three groups it was administered to, vincristine (Figure 1B) reduced the mechanical allodynia score by 61–65% (day-7 scores range between 1.8 ± 0.18 g and 2.0 ± 0.24 g vs. day 0 = 0.7 ± 0.07 g for all; *p* < 0.0001). In contrast, the control group that received the solvent alone showed no change in allodynia during these 7 days. PS treatment of mice with established vincristine-induced peripheral neuropathy for 16 days markedly improved allodynia scores (114% increase compared to day 0; *p* < 0.0001), with their score being identical to that of the control group (no vincristine). The difference between the PS-treated group and its vehicle control was statistically significant on day 16 (PS = 1.5 ± 0.09 *g*, vehicle = 0.8 ± 0.09 g; *p* < 0.0001). There was no appreciable change in allodynia scores during the same period in the vehicle or vincristine alone groups (0.7 ± 0.07 *g* vs. 0.8 ± 0.09 g for both).

As expected, during oxaliplatin administration (as described in the Methods), the allodynia score was significantly reduced (*p* < 0.0001) by 65 and 56% in the two study groups at day 0, respectively, (Figure 1C). PS treatment restored allodynia scores to the day 15 to the normal baseline (1.8 ± 0.09 g vs. 1.76 ± 0.13 g) whereas the vehicle group continued to show suppressed allodynia scores, being 47% lower on day 22 compared to day-15. The difference between the PS- and vehicle-treated groups became statistically significant on day 22 (*p* < 0.001).

**Figure 2:**
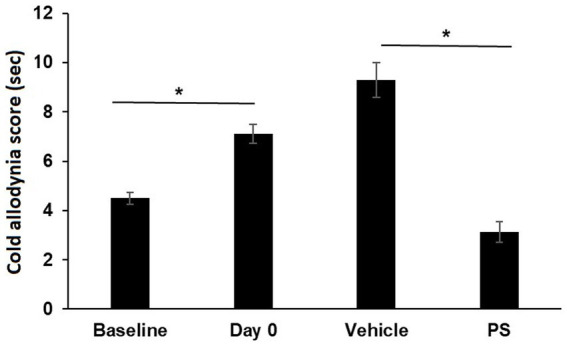
The effect of PS on cold allodynia. Two groups of mice were treated with paclitaxel for 7 days as in Methods for the induction of CIPN. Cold allodynia was measured by acetone test as in Methods. The cold allodynia score was the total time in sec spent licking/lifting/or shaking of the hind paw during a 1 min test period. Treatment of PS or vehicle gel applied to the hind paws 3×/d × 17 d started on day 0. Cold allodynia was determined before (baseline) and one day after the last dose of paclitaxel (day 0) and also on day 17 when the treatment with PS or vehicle was completed. One-way ANOVA Results: Group (*F*(3, 92) = 34.13, *p* < 0.0001). All values: *mean ± SEM*; n16 in paxlitaxel group and *n* = 8 in PS and Vehicle Treated groups. *p < 0.0001.

PS also improved cold allodynia induced by paclitaxel, as shown in Figure 2. Paclitaxel significantly increased the cold allodynia score from the baseline of 4.5 ± 0.2 s to 7.1 ± 0.4 s (*p* < 0.0001). Treatment with PS markedly reduced the cold allodynia score to 3.1 ± 0.4 s, which was significantly lower than the value after paclitaxel treatment (*p* < 0.0001). In contrast, 17 days of treatment with vehicle failed to decrease the cold allodynia score, which continued to increase (9.3 ± 0.7 s) even after paclitaxel treatment was discontinued.

In summary, PS was able to relieve the decrease in mechanical allodynia scores in CIPN induced by three commonly used chemotherapy drugs (paclitaxel, oxaliplatin and vincristine). Likewise, PS was able reverse the changes in cold allodynia scores induced by paclitaxel administration.

### 3.2. Preventive efficacy of PS

We also evaluated the ability of PS to prevent CIPN by studying its effect on paclitaxel-induced CIPN. The corresponding clinical situation is that in which the anti-CIPN agent is administered starting prior to, or concomitantly with the chemotherapy. In this study too, we used as an end point mechanical allodynia.

**Figure 3:**
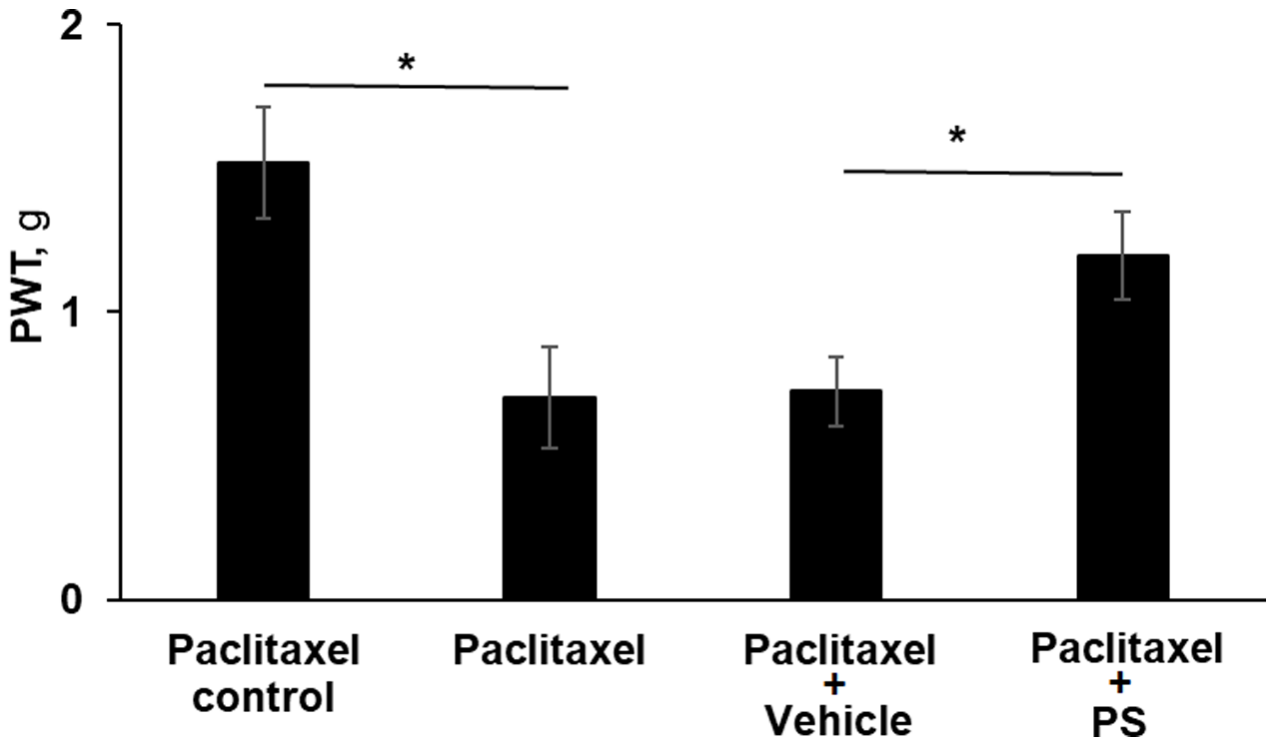
PS prevents CIPN mechanical allodynia. Three groups of mice were treated with paclitaxel and one with its solvent control (*n* = 8/group) for 8 days as in Methods for the induction of CIPN. Two of the paclitaxel treated groups had either PS gel or vehicle gel applied to their hind paws (3×/d × 10 d) beginning two days prior to the first injection of paclitaxel. Mechanical allodynia was determined manually with the von Frey test on day 10 (prior to euthanasia) and expressed as the PWT as measured in g. One-way ANOVA Results: Group (*F*(3, 21) = 39.87, *p* < 0.0001). *p < 0.0001, *n* = 6 to 7 mice per group.

As shown in Figure 3, three of the four study groups of mice were treated with paclitaxel (one injection every other day for a total of four) while the fourth group received the solvent alone, serving as control. Two of the paclitaxel-treated groups were pre-treated topically with either PS gel or vehicle (gel alone) 2 days prior to the first dose of paclitaxel. At the end of the study (day 10), the mechanical allodynia score of the paclitaxel group was 53% lower than its control (0.7 ± 0.17 g vs. 1.5 ± 0.19 g; *p* < 0.0001). The allodynia score of the vehicle-treated group was identical to the paclitaxel only group (0.7 ± 0.12 vs. 0.7 ± 0.17). However, the allodynia score of the pre-treated PS group was significantly increased compared to the vehicle group (1.2 ± 0.15 g vs. 0.7 ± 0.12 g; *p* < 0.0001), bringing it close to that of the paclitaxel control (1.5 ± 0.19 g). Therefore, like its effect on treating paclitaxel CIPN, PS could also prevent CIPN when its administration was initiated prior to the administration of chemotherapy and continued in parallel with it.

### 3.3. Dose response study

**Figure 4:**
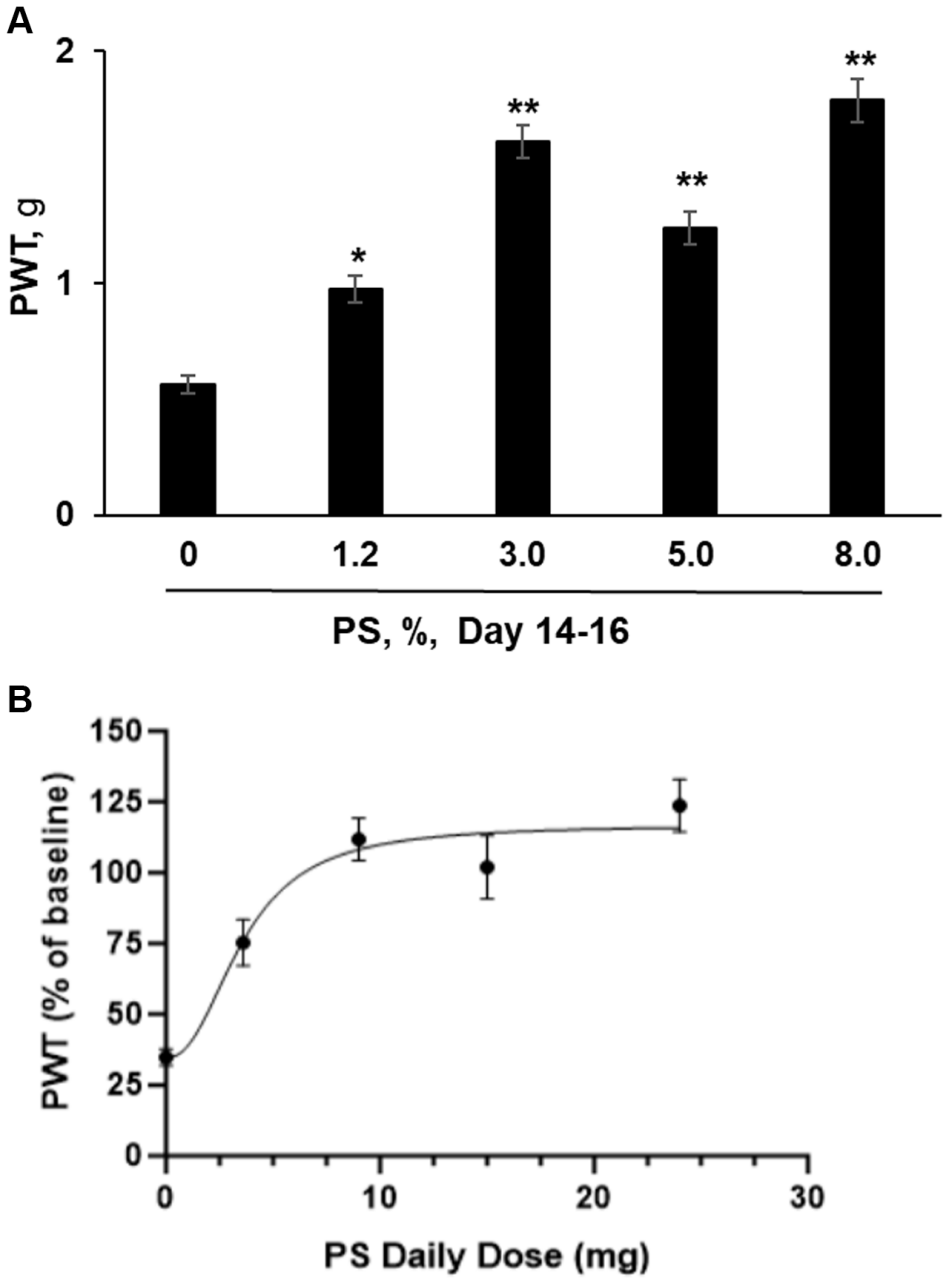
PS dose response study. **(A)** CIPN was induced in five groups of mice (*n* = 8-10/group) with paclitaxel as in Methods; a sixth group received the vehicle of paclitaxel (control) and developed no CIPN. Treatment of the CIPN groups with PS gel at the concentrations shown or vehicle ([PS] = 0) gel applied to the hind paws 3×/d × 14–16 d started on the day after the completion of treatment with paclitaxel. Mechanical allodynia expressed as the paw withdrawal threshold (PWT) and measured in g, was determined manually by the von Frey test. The results shown were obtained at the conclusion of treatment (day 14–16). One-way ANOVA Results: Group (*F*(4, 37) = 33.34, *p* < 0.0001). All values: *mean ± SEM*. *p < 0.005 and **p < 0.0001, compared to vehicle (0%). (**B)** The PWT as measured after 14 to 16 days of PS administration (expressed as a % of untreated baseline) was graphed verses the daily dose of PS in mg. A curve was drawn (see Material and Methods) using an equation for a four parameter nonlinear fit with variable slope. The Bottom and Top plateaus were calculated as 34.71% and 116.9% of baseline. The Hill slope value was 2.326 and the calculated EC_50_ value was 3.596 mg of PS per day. *R*^2^ was calculated as 0.4552. PWT was measured in both the right and left paw of each mouse. The number of mice measured at each dose was 8 except for the mice treated with 9 mg/d, where *n* = 10.

We also explored whether the effect of PS on CIPN in this animal model depended on its dose, using as an endpoint mechanic allodynia. CIPN was induced with paclitaxel as evidenced by the reduction of mechanical allodynia from 1.81 ± 0.13 at baseline to 0.54 ± 0.03 after its last dose. As shown in Figure 4A, we evaluated five concentrations of PS, 0, 1.2, 3, 5 and 8% on CIPN induced by paclitaxel. After 14 to 16 days of treatment, PS improved the allodynia scores in a dose-dependent manner, and the improved allodynia scores were 0.98 ± 0.06 g for 1.2%, 1.61 ± 0.07 for 3%, 1.24 ± 0.07 g for 5%, 1.79 ± 0.09 g for 8%. The improvement in allodynia scores by each dose of PS is significantly different when compared to vehicle alone (0.56 ± 0.04 g; *p* < 0.005–0001).

In Figure 4B we graphed the paw withdrawal threshold after 14 to 16 days of dosing with PS expressed as a percent of the baseline (measured prior to paclitaxel exposure) versus the daily administered dose of PS in mg. The calculated values of the four parameters as defined in the equation presented in the Materials and Methods are as follows for Top, Bottom, and Hill Slope, were: 116.9% of baseline, 34.71% of baseline, and 2.326, respectively. The fourth parameter, the EC_50_ value was calculated to be 3.596 mg/d. Because of the scatter in the data, the software returned only a lower limit of 1.550 mg/d for the calculated EC_50_ value.

### 3.4. Sulindac has no effect on CIPN

Various NSAIDs have been evaluated as potential treatments of CIPN, but to date there has been no evidence to support their oral administration to treat neuropathic pain conditions (Moore et al., 2015). The NSAID sulindac, the basic building block of PS, represents its main metabolite (Xie et al., 2012). Therefore, we compared the efficacy of sulindac 0.7%, the maximum concentration obtainable in the formulation used for PS, to that of its equimolar concentration of PS, 1.2%; they were administered in parallel to mice with paclitaxel-induced CIPN as above.

CIPN was induced in mice by paclitaxel as previously described and animals were treated with vehicle gel, or sulindac 0.7% or PS 1.2% 3x/day for 14 days, following the same protocol and using mechanical allodynia as the endpoint. At the end of the study, the allodynia scores were as follows: paclitaxel alone group = 0.61 ± 0.04 g; paclitaxel plus vehicle = 0.56 ± 0.04 g; paclitaxel plus sulindac = 0.62 ± 0.05 g (NS vs. paclitaxel); PS 1.2% = 0.98 ± 0.06 g (*p* < 0.001 vs. sulindac, vehicle, and paclitaxel). Unlike PS, sulindac failed to affect the decreased mechanical allodynia scores and thus has no effect on paclitaxel CIPN, in agreement with clinical observations (Moore et al., 2015).

### 3.5. PS administered to treat CIPN does not abrogate the efficacy of concomitant chemotherapy

Given the potential of PS to prevent CIPN, we examined whether it interferes with the anticancer efficacy of concomitantly administered chemotherapeutic agents. To assess this clinically relevant question, we studied the effect of topically applied PS on the anti-cancer efficacy of paclitaxel in the growth of murine Lewis lung carcinoma xenografts.

Paclitaxel 12 mg/kg was given for five consecutive days by i.p. to mice with Lewis lung carcinoma subcutaneous xenograft tumors, starting when their average volume was 188 mm^3^. As expected (Herbst et al., 1998), paclitaxel reduced tumor volume and at the same time induced CIPN. On the other hand, concomitant treatment with PS reversed CIPN but did not affect the anti-tumor efficacy of paclitaxel.

**Figure 5:**
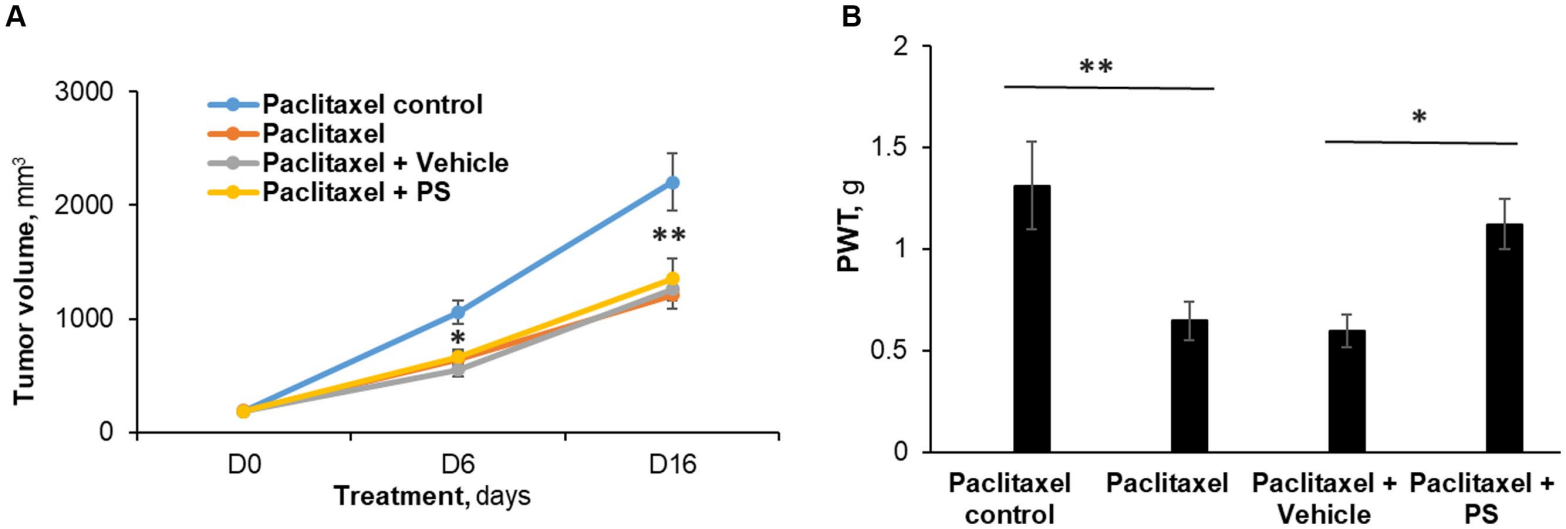
The efficacy of PS on allodynia during chemotherapy. **(A)** Mice with murine Lewis lung cancer (LL/2) subcutaneous xenografts (*n* = 8-10/group) were treated as shown for 7 days starting when the tumors reached ~180 mm^3^. Tumor size was measured at days 6 and 16 (end of study) of PS or vehicle treatment. Mixed Model results: Group (*F*(3, 29) = 13.34, *p* < 0.0001); Time (*F*(2, 58) = 258.14, *p* < 0.0001); Group*Time (*F*(6, 58) = 9.03, *p* = 0.0001). *p < 0.001 and **p < 0.0001compare tumor volume between paclitaxel treated mice verses paclitaxel control (receiving saline injections). *n* = 8–9 mice/group. **(B)**. Mechanical allodynia was determined manually with the von Frey test on day 7 and was expressed as the PWT measured in g. One-way ANOVA Results: Group (F(3, 29) = 5.04, *p* < 0.05).*p < 0.025, **p < 0.01, *n* = 8–9 mice/group.

Figure 5A depicts these results. Specifically, at the end of the study (day 16), paclitaxel reduced the tumor size by 45%, i.e., from 2,201.5 ± 253.5 mm^3^ (control group) to 1,208.6 ± 123 mm^3^ (*p* < 0.0001). In animals that, in addition to therapeutic paclitaxel, were treated topically with either PS gel or vehicle gel, the tumor volumes were essentially the same (PS = 1,351.9 ± 174.5 mm^3^ and vehicle = 1,259.2 ± 100.4 mm^3^; both NS compared to paclitaxel alone and to each other).

Paclitaxel treatment induced allodynia (Figure 5B) in these mice, evidenced by markedly reduced von Frey scores (control = 1.31 ± 0.20 g vs. paclitaxel = 0.65 ± 0.09 g; *p* < 0.01). PS essentially eliminated the mechanical allodynia (1.12 ± 0.12 g; NS vs. control; *p* < 0.05 vs. paclitaxel). The animals treated with vehicle gel failed to show any improvement in allodynia scores (0.60 ± 0.08 g; NS vs. paclitaxel). Thus, while the chemotherapeutic effect of paclitaxel was not affected by PS administration, PS was still able to improve the decreased mechanical allodynia scores.

### 3.6. The suppressive effect of PS on neuroinflammation

Paclitaxel is known to increase the production of pro-inflammatory cytokines and chemokines and induce the recruitment, activation and accumulation of macrophages with the pro-inflammatory M1 phenotype (Fumagalli et al., 2021). Thus, we studied in cultured cells the effect of PS on the production of selected cytokines and on the polarization (activation) of murine macrophages.

#### 3.6.1. Suppression of cytokine production

In co-cultured murine neuroblastoma and microphage cell lines (Neuro-2A and RAW264.7) that mirror the inflammatory infiltrate of CIPN, we evaluated the effect of PS on the expression of four cytokines, IL-6, IL10, CXCL-1, and CXCL-2, all involved in CIPN (Brandolini et al., 2019).

**Figure 6:**
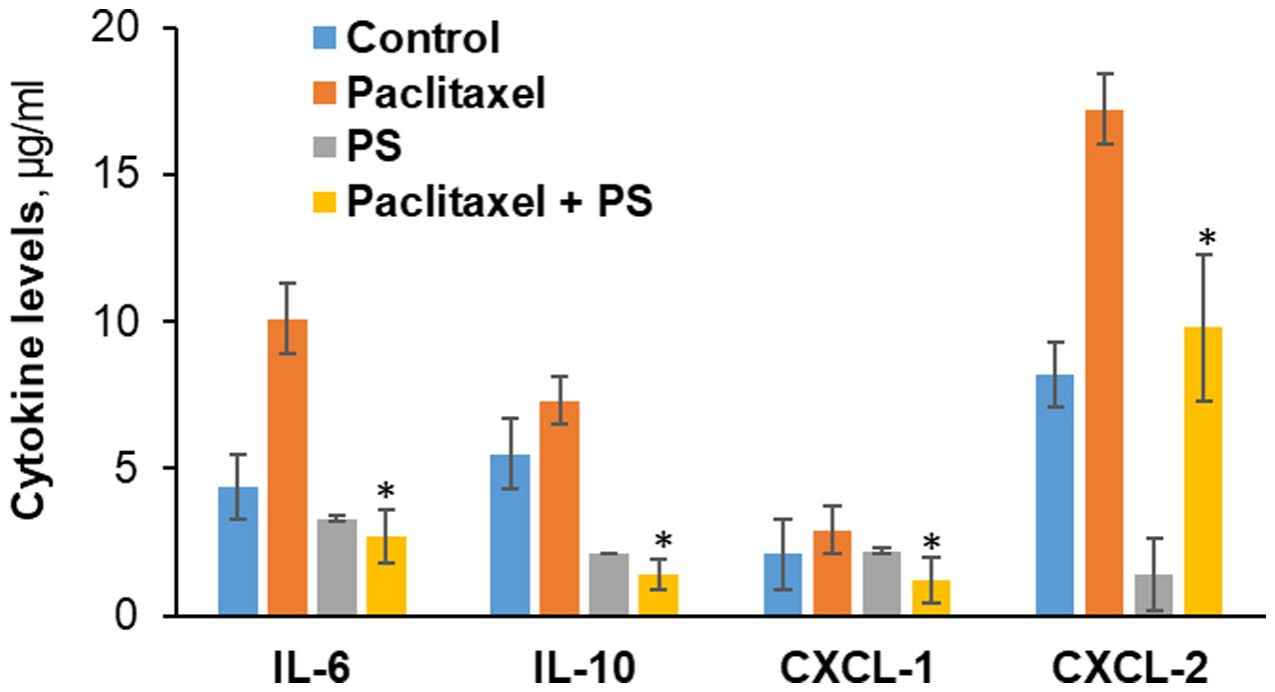
The effect of PS on cytokine levels in cultured cells. Neuro-2A murine neuroblastoma and RAW264.7 murine microphage cells were co-cultured as in Methods and treated with paclitaxel or PS or both for 24 h, when the culture media were harvested. The levels of the four cytokines shown were determined in the culture media using the magnetic bead-based immunoassay kit as in Methods. Values are the average of duplicates that were within 11% of each other. *p < 0.01 compared to paclitaxel.

As shown in Figure 6, paclitaxel significantly increased the levels of IL-6, IL10, CXCL-2 and only marginally of CXCL-1. PS significantly suppressed the paclitaxel-induced higher levels of all cytokines. Actually, PS suppressed the levels of the first three cytokines to below baseline, i.e., those of paclitaxel untreated cells. Except for CXCL-1, PS suppressed the unstimulated levels of cytokines as well.

#### 3.6.2. Suppression of macrophage activation

The proinflammatory M1 macrophages are an important source of cytokines in the neuroinflammation of CIPN (Ma et al., 2022; Msheik et al., 2022). *In vitro*, M0 macrophages can be activated (polarized) to the M1 phenotype using LPS and IFN-γ (Held et al., 1999).

**Figure 7:**
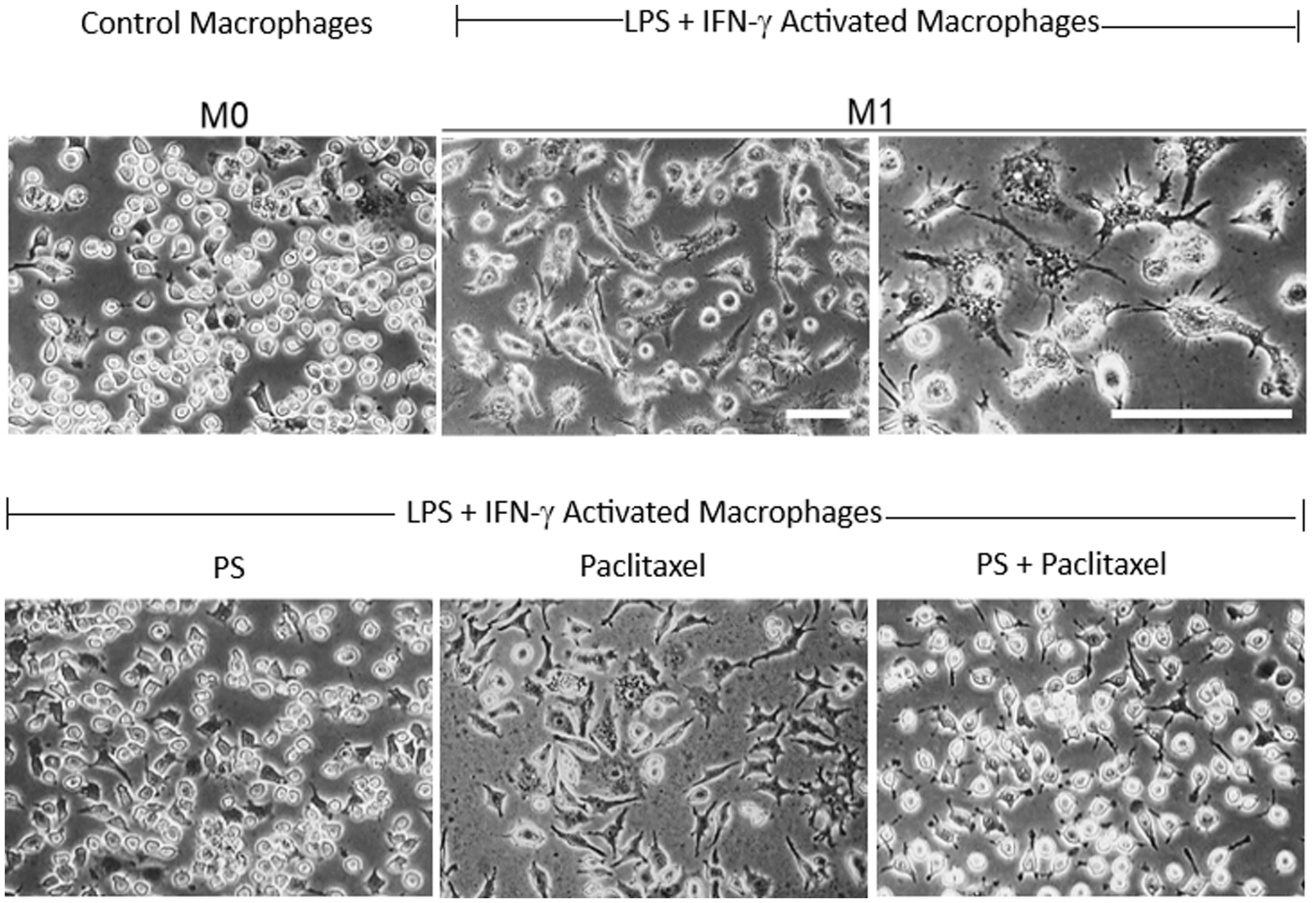
PS reverses paclitaxel induced macrophage activation. Upper panel: RAW 264.7 macrophages (M0; left) were activated after a 24-h treatment with LPS 1 μg/mL and IFN-γ 40 ng/mL (middle and left), incubated in control media, and photographed on day 4. Lower panel: RAW 264.7 macrophages activated as above, were treated on day 3 with PS (left) or paclitaxel (center) or both (right) and photographed on day 4. All photographs were obtained with inverted light microscopy under phase contrast. Magnification: 20X for all except for 40X in the third picture in the upper panel. Scale bars = 50 μm.

As shown in Figure 7, the RAW 264.7 macrophage cell line was activated to the M1 phenotype by LPS and IFN-γ, evidenced by the characteristic changes in their morphology. The M0 cells had plump, oval morphology, with dense cytoplasm, no vacuoles in most cells, nuclear borders not easily distinguishable from the surrounding cytoplasm, and only two or no pseudopods. In contrast, the M1 cells had generally flattened stellar shapes with multiple short cytoplasmic projections or lamellipodial extensions with thin ramifications and granular cytoplasm with several pale vacuolations in several cells, and nuclei that were clearly distinguishable from the cytoplasm with prominent nucleoli.

Treatment of activated macrophages (M1) with PS for 24 h essentially reversed their phenotype. In contrast, their treatment with paclitaxel did not have an appreciable effect on their M1 phenotype. However, when M1 cells were treated for 24 h with paclitaxel in the presence of PS 20 μM their phenotype was converted to the M0 inactivated non-polarized phenotype.

### 3.7. Therapeutic efficacy of the enantiomers of PS

**Figure 8:**
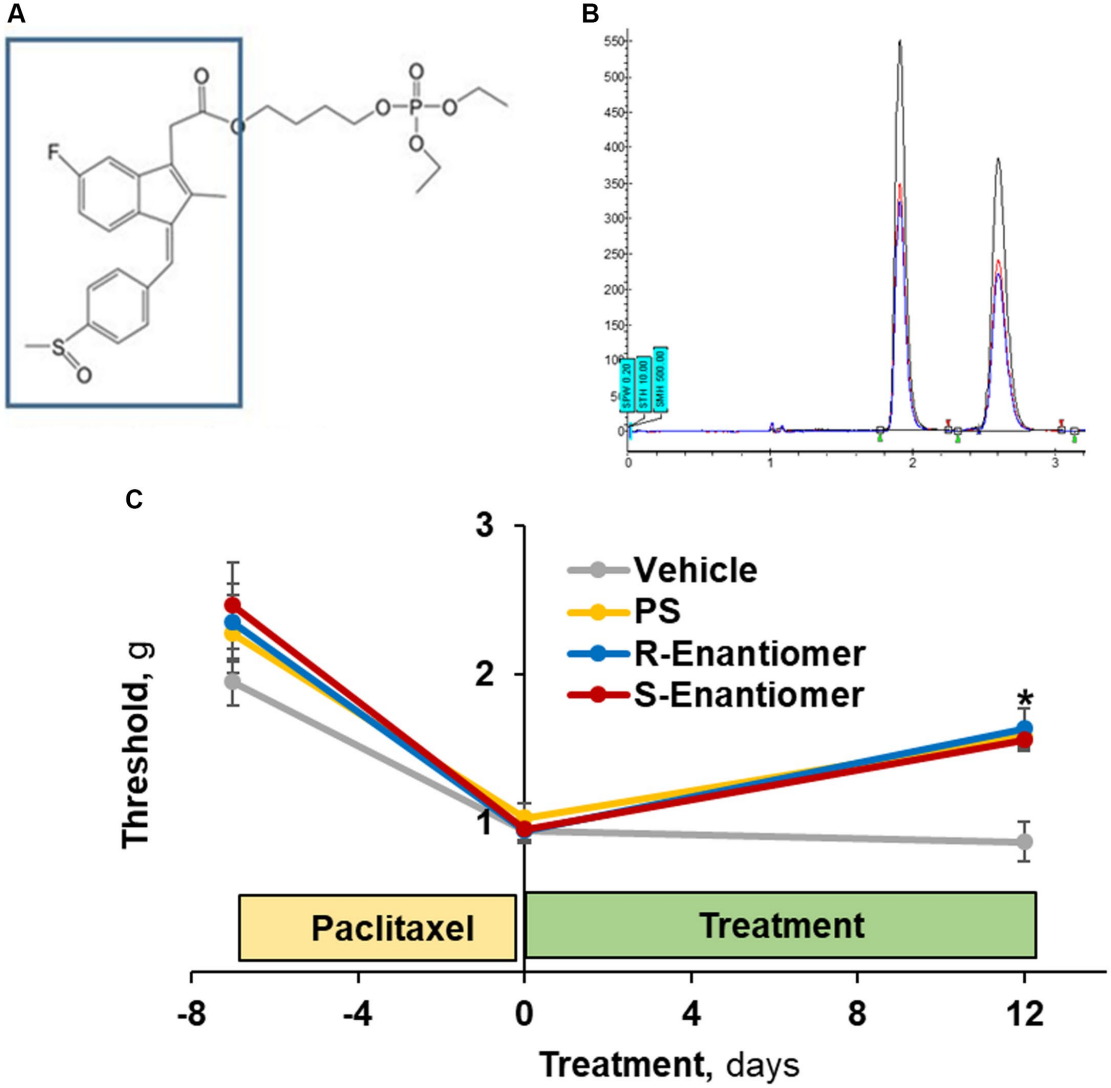
The efficacy of PS enantiomers in the treatment of CIPN mechanical allodynia. **(A)** The S atom of PS (the sulindac moiety in the box) is a chiral center. **(B)** Its two enantiomers were separated by supercritical fluid chromatography. **(C)** CIPN was induced in four groups of mice (*n* = 8/group) with paclitaxel as in Methods. When CIPN was established, mice were treated with either enantiomer, racemic PS (each as 5% gel) or vehicle, applied to the hind paws as above for 12 days. Mechanical allodynia was determined manually with the von Frey test and expressed as PWT measured in g. Mixed Model results: Group (*F*(3, 28) = 3.64, *p* < 0.025); Time (*F*(2, 56) = 43.96, *p* < 0.0001); Group*Time (*F*(6, 56) = 2.03, *p* = 0.077). Values: *mean ± SEM*. *p < 0.005 vs. vehicle.

The S atom of PS is a chiral center allowing for optical isomerism (Figure 8A). We isolated the two enantiomers of PS (Figure 8B), which were present in virtually equal amounts, and evaluated their efficacy on allodynia induced by paclitaxel.

To this end, we studied 4 groups of mice, all with paclitaxel-induced CIPN, evidenced by reduced scores of mechanical allodynia (average 56%; range: 51–61%). Treatment with either enantiomer (Figure 8C) as a 5% gel for 12 days after the induction of CIPN increased the allodynia score to a similar extent: S-PS: day 0 = 1.13 ± 0.14 g vs. day 12 = 1.64 ± 0.09 g (*p* < 0.005); and R-PS: day 0 = 0.96 ± 0.07 g vs. day 12 = 1.56 ± 0.18 g (*p* < 0.001). The racemate PS 5% gel had a similar effect: day 0 = 1.04 ± 0.09 g vs. day 12 = 1.59 ± 0.19 g (*p* < 0.005). Each of these effects was statistically different from that of the corresponding vehicle value on day 12 = 0.88 ± 0.08 g (*p* < 0.005 to 0.001), demonstrating that the enantiomers when compared to racemate PS are equally efficacious against allodynia.

### 3.8. The safety of PS

During all these studies, we did not observe any topical or systemic side effects of PS gel when applied thrice daily to the hind paws of mice for up to 22 days. This finding is in keeping with the known safety profile of PS (Cheng et al., 2012).

## 4. Discussion

CIPN remains a vexing and recalcitrant problem in cancer therapeutics. Its importance is underscored by its prevalence, spectrum of severity, and impact on cancer treatment outcomes and patients’ quality of life. The plethora of currently used chemotherapeutic agents has led to the reasonable suspicion that their diversity accounts to a significant extent to our lack of effective means to control CIPN. The range of proposed mechanisms of CIPN, some associated with specific classes of agents, supports this notion (Colvin, 2019; Eldridge et al., 2020). Developing the means to prevent or treat CIPN represents a pressing need.

Using a preclinical model of CIPN we have demonstrated the potential of PS, a topically applied small molecule to control CIPN. To date, PS has shown a broad range of anticancer and anti-inflammatory effects as well as a strong safety profile (Mackenzie et al., 2010; Huang et al., 2011b; Mackenzie et al., 2013).

The therapeutic effect of PS in established CIPN has several features that may have translational value. The response of allodynia, both mechanical and cold, was strong, in some cases returning to the pre-CIPN levels after a relatively short period of treatment. Allodynia is indeed a clinical hallmark of CIPN (Staff et al., 2017). The dose dependence of this effect is clear, supporting the notion of a strong pharmacological effect. The EC_50_ is 3.6 mg/d whereas an apparent plateau is reached at the higher concentrations (between 15 and 24 mg/d). More importantly, the three drugs that we tested (paclitaxel, vincristine and oxaliplatin) represent three of the commonly used classes of chemotherapeutic agents. In addition, they are structurally and mechanistically distinct in their anticancer activity. Furthermore, there is divergence in the mechanisms by which each is considered to induce CIPN (Sisignano et al., 2014). Whether PS’s efficacy extends to other CIPN-causing drugs is, however, uncertain, requiring further study.

Another clinically relevant scenario reflected in our studies is that PS maintained its anti-CPN effect while not diminishing the anti-cancer effect of concurrently administered chemotherapy (paclitaxel). If confirmed for additional chemotherapeutic drugs, it would be a clinically useful feature, as cancer patients with established CIPN often require additional rounds of chemotherapy.

In addition to relieving already established CIPN, PS can also prevent it. Under a chemo preventive neuropathy protocol, PS displayed a preventive effect that was both robust and rapid. Such a manner of PS administration could potentially evolve to a clinically useful modality and even the standard of care.

Chirality plays a fundamental role in the binding affinity and interactions between a drug and its target, thereby shaping its pharmacology (Brooks et al., 2011; Silvestri and Colbon, 2021). For this reason, the Food & Drug Administration (FDA) has issued guidelines for the pharmaceutical development of single enantiomers and racemates (Agranat et al., 2002). Thus it is important to evaluate the enantiomers early in drug development (Abram et al., 2019). Our results establish that PS is a racemate with its two enantiomers at essentially equal concentrations. Of interest, sulindac, its parent compound, is known to be a racemate (von Maltzan et al., 1998) and its enantiomers have been studied (Brunell et al., 2011). Our efficacy data document that the two enantiomers of PS are of equal efficacy, and the presence of each in the racemate does not affect the efficacy of the other. Thus, based on these data, it is likely that the racemate of PS merits further preclinical development.

CIPN is well suited for topical treatment because its clinical manifestations are limited to hands and feet (often not all of them) and all currently available agents, which are systemically administered, are associated with significant side effects (Loprinzi et al., 2020). A topically formulated drug traverses the epidermal tissue and increases the nociceptive threshold (Knezevic et al., 2017). The topical route provides low systemic clearance, reduced drug interactions, increased patient tolerability, and facile combination with oral medications (Sawynok, 2014). Our topical formulation of PS, the result of screening multiple permeation enhancers (Kovacik et al., 2020), displays these advantages.

An intriguing question concerns the mode of action of PS in CIPN. PS is hydrolyzed by the ubiquitous carboxylesterases to sulindac and its metabolites (Wong et al., 2012; Xie et al., 2014), thus raising the question whether PS behaves as a pro-drug, acting through sulindac or even through sulindac’s active metabolite, sulindac sulfide (Duggan et al., 1977). Our study with equimolar amounts of sulindac and PS in which only PS was efficacious against CIPN establishes PS as the active agent. This is consistent with our previous work with carboxylesterase knockout mice in which PS had much higher anticancer efficacy compared to wild type mice (Wong et al., 2015). A carboxylesterase inhibitor used in wild type mice generated similar results (Wong et al., 2012). Moreover, extensive human studies offer no evidence to support the use of NSAIDs to treat neuropathic pain including CIPN (Moore et al., 2015).

Our data also provide a glimpse into the mechanism of action of PS, establishing the suppression of cytokines. As already stated, cytokines are an important component of the neuroinflammatory response that characterizes CIPN.

The co-culture of nerve and macrophage cell lines that we employed attempts to recapitulate the *in situ* intense macrophage infiltration of nerves affected by CIPN (Kiguchi et al., 2017; Ma et al., 2022). The four cytokines that we assayed play a significant role in the pathophysiology of CIPN. For example, increased IL-6 activity is associated with painful CIPN (Starkweather, 2010); endogenous IL-10 is required for the resolution of CIPN-associated pain (Krukowski et al., 2016); and CXCL-1 (Cho et al., 2023) and CXCL-2 (Kiguchi et al., 2017) are significant contributors to CIPN.

The pattern we observed is that paclitaxel induces the expression of these four cytokines and PS reverses it. Thus, consistent with the role of these four cytokines in CIPN, we may conclude that PS ameliorates CIPN by interfering with neuroinflammation. A significant contribution to this effect of PS may come from its ability to reverse the activation of macrophages, which contribute to the neuroinflammation of CIPN by not only producing cytokines but also by further stimulating the entire process (Msheik et al., 2022). That we observed these effects in the presence of paclitaxel activation of macrophages suggests that such a mechanistic effect is plausible *in vivo*.

In summary, our results characterized the pharmacological action of PS against CIPN showing, among others, its broad therapeutic and preventive efficacy against CIPN. Collectively, our findings underscore the translational potential of this topically applied small molecule that mechanistically appears to target neuroinflammation. These data support further evaluation of topical PS for the control of CIPN.

## Data availability statement

The raw data supporting the conclusions of this article will be made available by the authors, without undue reservation.

## Ethics statement

The animal study was approved by the Institutional Animal Care and Use Committee of Stony Brook University. The study was conducted in accordance with the local legislation and institutional requirements.

## Author contributions

DB, LH, and BR: participated in research design. AB, JY, VT, AL, DB, AD, AM, GK, RH, LH. MK, NF, EW, AJ, and SS: conducted experiments. BR: contributed new reagents or analytic tools. DB, AD, AM, GK, RH, BR, EN, and LH: performed data analysis. AB, VT, DB, EN, and BR: wrote or contributed to the writing of the manuscript. All authors contributed to the article and approved the submitted version.
